# Exploring Cultural Humility as a Framework for Supervising Second-Language Nursing Students Writing Their Bachelor’s Thesis: A Qualitative Study

**DOI:** 10.3390/nursrep16070229

**Published:** 2026-07-01

**Authors:** Marte Bodil Roed, Monica Nilsen Moerch, Nina Beate Hamre, Marianne Loennebotn

**Affiliations:** 1 Department of Health and Caring Sciences, Western Norway University of Applied Sciences, Inndalsveien 28, 5063 Bergen, Norway; monica.nilsen.morch@hvl.no (M.N.M.); marianne.lonnebotn@hvl.no (M.L.); 2 Department of Health and Function, Faculty of Health- and Social Sciences, Western Norway University of Applied Sciences, Inndalsveien 28, 5063 Bergen, Norway; nina.beate.hamre@hvl.no

**Keywords:** bachelor’s thesis, cultural humility, L2, Norway, nursing, nursing education, qualitative study, second language, supervision

## Abstract

**Background/Objective:** Colleges and universities worldwide are experiencing an increase in students from diverse cultural and linguistic backgrounds. Nursing students who study in a second language face both theoretical and practical challenges within their study programs. If these challenges are unaddressed, they may have implications for third parties such as employers, patients, and colleagues. The bachelor’s thesis challenges students’ understanding of a chosen subject within nursing, in addition to research skills and academic writing abilities. Effective student learning and final thesis results often rely on supervisors who are interested in and motivated by the students’ success. Thus, the aim of this study was to explore supervisors’ experiences and perceptions of supervising undergraduate second-language (L2) nursing students in their bachelor’s thesis work. **Methods:** A qualitative research approach was employed to collect and analyze data. Components of Systematic Text Condensation were used for sorting and coding, and Interpretive Description was applied in the interpretive analysis. **Results:** Four prominent conceptual themes were agreed upon by the researchers: (1) uncertainty and cultural barriers, (2) cultural considerations, (3) teaching strategies, and (4) supervisor integrity. The conceptualized themes were found to align with the five identified attributes of cultural humility; openness, self-awareness, egolessness, supportive interaction, self-reflection and critique. **Conclusions:** This study suggests useful insights and practical guidance to supervisors on central issues to consider and reflect upon when guiding nursing students who are writing their bachelor’s theses in a second language. The theory of cultural humility, including the identified attributes, appears to be a useful framework for supervisors when guiding L2 nursing students.

## 1. Introduction

Higher education institutions worldwide are experiencing an increase in the enrolment of students from diverse cultural backgrounds and various nationalities due to the changing trends in global mobility [1,2]. These students may face challenges of “triple socialization”, which refers to the process of being socialized into a new academic system, in addition to a new language and a new culture [3] (p. 27), [4,5,6]. Second-language (L2) students who pursue a bachelor’s degree in nursing face both theoretical and practical challenges within the study program [4,7]. The International Council of Nurses’ position statement on cultural and linguistic competence states that “…nurses should be culturally and linguistically competent to understand and respond effectively to the cultural and linguistic needs of clients, families and communities in a health care encounter” [8]. Various approaches, such as language groups and writing centers, have been implemented on university campuses globally to support students facing linguistic challenges, yielding varying results [4,9].

The bachelor’s thesis in nursing is meant to challenge students’ understanding of a chosen topic within the field of nursing, while also developing their research skills and academic writing abilities [10,11]. Students’ own perspectives on writing the bachelor’s thesis include achieving a higher understanding and knowledge of the nursing profession [10,12]. A recent scoping review examined how L2 undergraduate nursing students self-reported their academic success [7]. The findings indicated that students with higher levels of communication comfort reported greater academic success. Students also suggested that faculty should provide more detailed explanations of key points and concepts during teaching sessions and demonstrate greater sensitivity to diverse student needs [7].

Furthermore, important components of academic supervision in nursing education include enhancing students’ critical thinking and promoting nursing as a knowledge-based academic profession [13,14,15]. Integrating clinical practice with research and academic literacy also aligns with supervisors’ professional nursing identity [11]. Academic writing can be challenging for students writing in their first language, and often even more so for L2 students [16]. A study conducted in a Chinese setting examined undergraduate L2 students’ negative emotions during thesis writing and reported high levels of stress associated with the writing process. Identified stressors included engaging with complex academic texts, perceived negative feedback from supervisors, and the required length of the thesis [16].

A productive collaboration with the supervisor can be crucial for fostering effective learning and achieving good results [12,14]. A literature review on academic integration among nursing students for whom English is a second language found that support from culturally sensitive counselors with diverse backgrounds is an essential factors for students’ success [15]. In addition to providing academic guidance, supervisors take on roles such as counselors, educators, inspectors and communicators [14]. A recent study from Spain found that supervisors emphasize their roles as guides in students’ emotional management and as promoters of self-responsibility. Understanding the complexity and individuality of each student requires supervisors who are genuinely interested in and committed to supporting students’ success [17]. Another important task for supervisors is to provide effective and constructive feedback to students. This can be particularly challenging if students struggle with linguistic and cultural challenges, considering the institutional frames allocated to supervision [14]. Fear of the supervisor, or fear of the feedback given by the supervisor, may also cause the student to avoid contact with the supervisor [3] (p. 48).

### 1.1. Cultural Diversity Concepts

Various concepts and theories concerning cultural diversity started to emerge around the 1980s as an approach to understanding and caring for ethnic minority groups in the US [18]. The broadly used concept of cultural competence appeared as a welcome strategy for professionals in health and social sciences, as their user groups became more culturally diverse [18,19]. Cultural competence has been criticized for implying “expert knowledge about other cultures” [19], which has contributed to the evolution of other nuances of the concept. The overlapping concepts of cultural sensitivity and cultural safety claim to give more attention to the user groups of diverse cultures, with slight distinctions [18]. Kirmayer (2012) defines cultural safety as a systemic approach for mitigating inequalities and power differences within health systems, whereas cultural sensitivity entails listening to and learning from patients through interaction [18]. More recently, the theory of cultural humility was initiated and developed by Cynthia Foronda (2020) as a guide to approach and manage cultural diversity [20]. The theory was built on a foundation of Leininger’s cultural care theory [21], and the cultural humility term was adopted from Tervalon and Murray-Garcia [22]. The concept analysis undertaken by Foronda et al. (2016) explains how the term cultural humility has emerged from other terms such as diversity, cultural competence, and cultural sensitivity [23]. Foronda et al. [23] also suggest that cultural humility should be highlighted and applied within all areas of professional work, as it emphasizes the lifelong process of learning skills through identified attributes of openness, self-awareness, egolessness, supportive interaction, self-reflection and critique. “Cultural humility does not focus on competence or confidence and recognizes that the more you are exposed to cultures different from your own, you often realize how much you don’t know about others. That is where humility comes in” [24]. A study conducted by Mrayyan [25] found that nursing students perceived their educators as having low scores on cultural humility.

### 1.2. Justification for the Study

In Norway, everyone who qualifies has the right to access higher education, which creates a student body of great diversity in socioeconomic backgrounds and motivational factors [26]. Foreign students must pass standardized language requirements in written and oral Norwegian and English (B2 level), in addition to the general study competence diploma [27]. Research reveals that although formal language requirements are met, it does not always reflect the students’ academic performances [28]. Equality in admission criteria and supervision guidelines does not necessarily ensure equity among the student population. Denham et al. [7] emphasize the importance of awareness and reflective practices among academic staff regarding inclusive strategies and teaching practices for diverse student groups. For L2 students, culturally sensitive and adept supervisors are essential for students’ academic success rate [17]. Various studies describing the general supervisor–student relationship, as well as student satisfaction and success, have been published [7,10,29]. However, structured literature searches in scientific databases have revealed a lack of research on supervisors’ experiences and perceptions of supervising L2 nursing students in their bachelor’s thesis work. To address this gap, the aim of this study was to explore supervisors’ experiences and perceptions of supervising undergraduate L2 nursing students in their bachelor’s thesis work.

## 2. Materials and Methods

The study is situated within the interpretivist constructivist paradigm using an inductive phenomenological approach. According to Mckenzie and Knipe [30], phenomenology is embedded in the interpretivist constructivist paradigm. We used qualitative methods to explore in-depth supervisors’ experiences and perceptions of language and culture. Subsequently, we analyzed and interpreted the data to understand how the supervisors apply this knowledge in their supervision of L2 nursing students. For sorting and initial analysis of the data, we applied components from Systematic Text Condensation (STC) [31,32] (pp. 118–129). Interpretive Description guided the applied and conceptual interpretation (ID) [33]. Adhering to ID, we aspired to merge data collected from the practical disciplines of nursing and teaching, with interpretations of the findings reaching into philosophical and social sciences [33] (p. 18), to advance the practical implications for supervision of L2 nursing students.

### 2.1. Information and Recruitment

Information about the planned study project was presented to attending supervisors at three staff meetings at a university in Norway in June and August of 2023. Purposeful recruitment of eligible supervisors was attempted at a university department gathering in February 2024, with follow-up information about the study via e-mail. Information about the study and invitations to participate were also sent via e-mail to all supervisors of bachelor’s theses in nursing at four university campuses in Norway, approximately 6o people in total. The inclusion criteria were experience with bachelor’s thesis supervision for students with Norwegian as a second language and that the supervisors had an educational level of master’s degree or higher.

### 2.2. Participants and Data Collection

Seven supervisors responded via e-mail and signed up for participation in the study. None dropped out during the study period. All data was collected in the time period from April to September 2024. Both individual interviews and focus group discussions (FGDs) were conducted, as variation of methods may attain more nuanced perspectives and diverse responses from the supervisors [33] (p. 118). One FGD with three supervisors was moderated by the researcher NBH, and four individual interviews were conducted by either NBH or MNM. The researchers did not have any previous acquaintance with the supervisors interviewed. All interviews were held face-to-face, except one interview that occurred digitally via Zoom due to a long travel distance. A semi-structured interview guide containing three openly phrased questions was developed and discussed among the researchers prior to the interviews (Appendix A). Following recommendations of Thorne (2025) [33] (pp. 116–117), the main questions were supplemented with prompting from the researcher to retrieve more nuanced or elaborated responses. The interview guide was not pilot-tested due to the flexibility of the researcher to amend and clarify questions during qualitative interviews, and no repeat interviews were conducted. Quiet and secluded meeting rooms at university campuses were used as locations for the physical interviews and FGDs. All interviews and FGDs were recorded and had a mean duration of 40 min. Some notes were taken for clarification. The recordings were subsequently transcribed verbatim by the researcher MBR. The sample specificity in this study was dense, with the majority of the participants having substantial supervision experience. A narrow study aim combined with a diverse sample and a strong dialogue in the interviews provided high data richness. Hence, the information power [34] in the data material was assessed to be high. Transcripts were not returned to participants for verification.

### 2.3. Data Analysis and Interpretation

In ID, drawing inspiration from existing coding methods is considered beneficial for organizing the data [33] (p. 136). Thus, the analysis process was initiated by adhering to the first two steps of the STC method by Malterud [32] (p. 117). In step one, all the transcribed data material was read individually by each researcher to gain a general overview of the material and suggestions of preliminary themes. The preliminary themes were then discussed in several physical meetings until consensus was reached among the researchers. In step two, the transcribed documents were uploaded to the software program NVivo 15.0 for systematization of meaningful text units into different color-coded groups corresponding with the preliminary themes.

Adhering to the methodology of ID [35], the data material, themes and code groups were consistently compared and analyzed during the data collection period. All coded text extractions were then thematically combined into separate Word documents and re-read by all researchers to quality-check and adjust the coding across the thematic documents. Continued analysis consisted of reflexive interpretation and conceptualizing of the phenomenon of interest, concordant with ID [33] (pp. 164–168). To illuminate the phenomenon of interest in the most meaningful way, ID suggests drawing upon interdisciplinary theories in the analysis process to shed new light on established social contexts [33] (p. 195). The researchers reviewed existing literature related to “cultural diversity” for explanatory insight as the analytical process evolved [35,36]. Trustworthiness was obtained by combining the subjective experiences of the interviewees with conceptualizing reflexivity and discussions among the researchers [33] (pp. 209–211). As the researchers submerged themselves in various concepts of cultural diversity and reflexive discussions around the phenomenon, the concept of cultural humility appeared to be the most suitable theory connecting with the coded data. During the interpretation process, the data was arranged into an analysis and interpretation table, as exemplified in Table 1.

### 2.4. Reflexivity and Roles of Researchers

Identifying one’s own biases and preconceptions when conducting research within one’s own work environment is generally regarded as critical for creating a valid and trustworthy product [36]. However, in ID, rather than seeing researchers’ experiences and contextual knowledge as obstacles, these resources are seen as strengths and insights to better understand and analyze the collected data [33] (p. 238). In this study, all researchers have experience with supervision of L2 students. The researcher NBH (MPhil) is an occupational therapist and assistant professor of occupational therapy. MNM (M.Sc.) is a licensed nurse midwife and assistant professor with research experience from both clinical settings and teaching. ML (PhD) is a licensed nurse midwife and associate professor in midwifery. She provided practical guidance and supervision during the study period. The main author, MBR (MPhil), is a licensed nurse and assistant professor in nursing, with educational and research background from international contexts. Doing research within any field of science requires modesty and responsible conduct by the researcher [33] (p. 112). The two researchers who conducted the interviews (MNM and NBH) had previous experience and training in qualitative interviewing techniques. The study was conducted within the researchers’ own context of institutional structures in higher education, which makes self-criticism essential when interpreting participants’ responses.

### 2.5. Ethical Considerations

All participants were given an invitation letter describing the purpose of the study with an attached consent form based on the template from the Norwegian Agency for Shared Services in Education and Research (SIKT) [37]. Signed consent was obtained from all participants, who were also provided the opportunity to withdraw from the study at any time. All collected information was kept digitally in double-password-protected computer programs, with personal authentication keys. Data will be deleted by June 2030. The COREQ checklist was used to ensure ethical principles through the research process [38]. The study received ethical approvals from the Research Ethics Committee at Western Norway University of Applied Sciences (HVL), and from SIKT (Reference number: 348597).

## 3. Results

The supervisors interviewed were all university teachers in a Bachelor of Nursing Science program at a Norwegian university, with various clinical and academic backgrounds. Some of the participants were L2 supervisors with educational backgrounds and work experience from other countries. Table 2 gives an overview of the participants.

**Figure 1:**
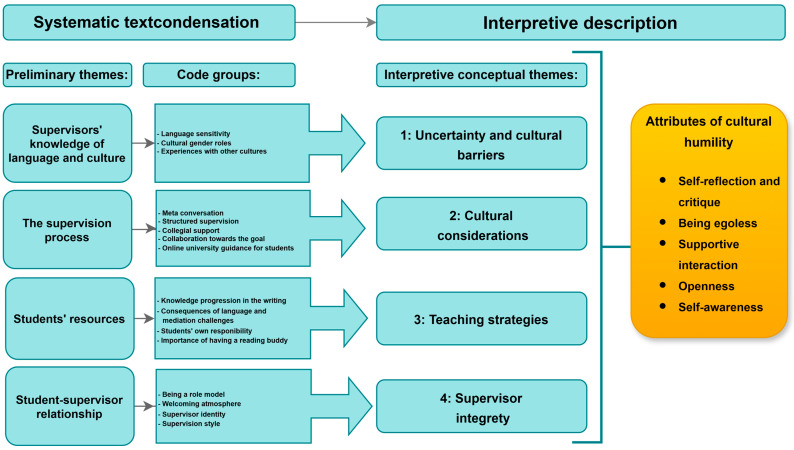
Conceptual interpretation aligned with attributes of cultural humility [23].

Four prominent conceptual themes were agreed upon by the researchers and categorized as: (1) uncertainty and cultural barriers, (2) cultural considerations, (3) teaching strategies, and (4) supervisor integrity. The conceptual themes aligned with the attributes of cultural humility [23], which complemented and elaborated on the existing findings (Figure 1). The findings revealed that a majority of the supervisors regarded experience with other cultures and languages as an important factor for providing individualized supervision to L2 students. Maintaining awareness of one’s own identity and adopting a curious and humble approach towards L2 students were believed to uphold the integrity of the supervisors.

### 3.1. Uncertainty and Cultural Barriers

Most participants contended that having a history of exposure to diverse cultural settings was relevant for their role as supervisors of L2 students. Some supervisors had worked with multicultural students in contexts such as exchange programs, whereas others had experiences from working in foreign countries, which they claimed had broadened their understanding of cultural differences:

“*I have lived many years of my life abroad, so I know something about having to express myself in a language that isn’t my own. So, I think that’s perhaps what I connect with when I meet these students. And sure, it’s about language, but it’s also much about culture, and seeing things in a different way, or having the opportunity to have your own identity.*”

(Associate professor 4)

Some of the supervisors highlighted that a certain knowledge and experience with L2 students made them more capable of detecting “the great value that lies there” (Associate professor 5), beyond the poor language skills. Supervisors who did not have experience with diverse cultures contended that they may attribute obstacles in supervision to language barriers only, without considering other cultural differences, as put into words by one supervisor:

“*I always get a bit unsure, when talking to L2 students, about where exactly the challenge lies. Is it that I’m not conveying it well enough to them, or is it that they don’t accept it, or is it perhaps a lack of knowledge? (laughs). And maybe… sometimes we might be a bit quick to address it to a lack of knowledge, instead of thinking; what is it… are there any language barriers here that are causing this…? Or maybe they have a different body language that means they actually understand it, but they respond in a way that I don’t expect, you know.*”

(Assistant professor 7)

Several of the supervisors mentioned perspectives of cultural differences in gender roles, particularly in the context of professional relationships and supervision on the bachelor’s thesis. Three of the supervisors talked about how they had experiences with male students who found it difficult to take guidance from them as female supervisors, especially if the supervisor was younger than the student. One supervisor explained how she used a strategy of talking openly with the students and sometimes asked them directly if they found it difficult to collaborate with her. The students would rarely admit openly that they found the gender dynamic difficult. Nevertheless, addressing the issue often created an awareness of the topic, which gave both the supervisor and the student opportunities for further reflections on cultural differences. It also opened discussions on how to proceed in the student–supervisor collaboration.

### 3.2. Cultural Considerations

The supervisors were asked what they think is of utmost importance when guiding L2 students in academic writing. Without exception, all supervisors acknowledged the importance of having an individual start-up dialogue, or a meta-conversation, before discussions about the bachelor’s thesis. Having a meta-conversation entailed agreeing on common goals and establishing ground rules for a collaborative relationship between the student and the supervisor. Several of the supervisors also admitted to giving the students an extra session, on top of the established number of sessions allocated in their worksheets, as one supervisor declared:

“*Maybe that extra hour you give, which is free in a way, saves you so much time later on, you have no idea, yes, it’s more about getting to know the student than getting to know the culture or language, right.*”

(Associate professor 6)

Creating a safe and trustworthy environment in consecutive supervision sessions was also important for the supervisors, and they often initiated a session with small talk, as this was emphasized as an important warming-up strategy. Sometimes the students revealed personal struggles with family issues, children and part-time jobs, which made it difficult to focus on writing the thesis. When the students experienced understanding and empathy from the supervisor, it often released some tension. The students seemed to become more at ease, which contributed to better mutual collaboration towards the goal. Many of the supervisors also emphasized the principles of equality and structure, aiming to provide equal supervision and opportunities to all students. This was exemplified by suggesting strategies and tips for improving students’ written language, regardless of the students’ proficiency, as the following extracts from the focus group discussion show:

“*I make sure they [L2 students] get the same offers as other students and that they are informed about the digital tools the university has. For example, the library, various software they can download, getting connected with the right departments that can help them with this, for example IT, right.*”

(FGD, Assistant professor 1)

Another supervisor wished for more structured digital information about existing opportunities and viable tools that the university had to offer to assist students with language challenges, such as writing groups and proofreading software.

Supervisors with personal backgrounds from countries other than Norway highlighted the differences in educational cultures. Academic institutions with a more defined hierarchical system between students and supervisors could put restraints and limits on the student’s ability to engage in creative and reflexive thinking in academic writing:

“*This hierarchy, where the teacher says this is what you should do […] Yes, students are not used to being asked ‘What is your idea? What is your plan?’ So that is something to consider in supervision regarding culture. We need to be more aware that students need guidance. That they can, or they must—contribute. Take responsibility.*”

(FGD, Associate professor 2)

Sometimes unwanted and difficult situations would arise during the supervision period due to cultural differences. If the supervisors encountered problematic issues in some ways, they often sought advice from other colleagues about alternative ways to approach a situation. Several supervisors also wished for an arena for discussing cultural and ethical issues related to supervision of L2 students, as cited below:

“*I have many experiences that I have accumulated myself, but they are private, and I’m sure other people have them too. So a bit of experience exchange among colleagues should have been on the agenda.*”

(Associate professor 5)

### 3.3. Teaching Strategies

Although many supervisors were willing to “go an extra mile” to assist L2 students, they also had high expectations and anticipated good effort and hard work from all students during the thesis writing period. If the students did not show extensive knowledge about their chosen topic, the supervisor would guide the students to use the library resources for further study and reflection. Sometimes inconsistencies occurred in expectations of the quality of the thesis, where ultimately it was the student’s decision on how to proceed:

“*Oh yes, [it happens] many times. But then I go back to my rules, you know. ‘Do you think this is important? Okay, well, write about it; why do you think so…? But again, it’s their assignment, not my assignment, I always say that. I already have a bachelor’s degree.*”

(Associate professor 6)

Another supervisor considered whether it would sometimes be beneficial for some students to fail on a low-quality paper, as this would give them the opportunity to resubmit the thesis and receive additional guidance, which may yield better results. Generally, when referring to low-quality papers, the supervisors were concerned with the curricular content in the paper, not the language or grammar:

“*I base on what they write, and then I push them a bit further. ‘What does this mean? What is behind this? Can you explain it?’ […] In other words, analytically. I don’t use that word, but what is it about? Very quickly, our academic language stands in the way of understanding. It becomes so alienating.*”

(Associate professor 5)

A united recommendation from the supervisors to the L2 students was to obtain a reading buddy who could provide feedback on their thesis document, not on the curricular content, but as a lay person reading Norwegian. One supervisor drew similarities between students with language barriers and students with dyslexia, based on L2 students’ feedback about their experience from writing in Norwegian: “*They hear it in their head how it should be, but they can’t get it down on paper like that.*” (Assistant professor 7)

Notwithstanding the language barriers, most of the supervisors stressed the great resources the L2 students contained within themselves, based on their various background experiences, which could include fleeing from another country, growing up in other cultures, and the process of adapting to Norwegian culture and the educational system, as one teacher proclaimed:

“*I definitely think that they are used to having to work on things, even more than the Norwegian students. Yes, and of course there are many exceptions, but many of the hardest working students are those who have Norwegian as a second language.*”

(Associate professor 4)

The supervisors all agreed that breaking the hidden codes of a new language and unfamiliar culture was no small task, but it could yield great benefits for those who succeeded. Linguistic and academic progression go hand in hand, but can happen in stages, as identified by one supervisor:

“*You also have that layer of language on top, right? I mean, what have they understood at each stage of their education? Because you have the academic development, and I also think there can be language development over the course of three years.*”

(Assistant professor 7)

### 3.4. Supervisor Integrity

Sometimes supervisors faced their own preconceptions in relation to guidance of L2 students. One supervisor explained how she may initially be biased towards students who spoke poor Norwegian, assuming that they would also perform poorly, but often had to reconsider. Taking time to listen to the students to assess what abilities the students had within them, especially regarding theoretical concepts, would often yield good results. Hence, being open-minded and conscientious in meetings with students was important:

“*What do we need as supervisors, and what do we need as human beings? Because it is not only in the supervision situation that we need to reflect on who… which people we are meeting. And that applies not only to L2 individuals, it applies to all types of people. So I actually think that for us as supervisors, as human beings, as teachers, you must have some fundamental values, in meetings with people.*”

(Assistant professor 4)

One supervisor who had Norwegian as her second language reflected on the importance of her own supervision style when collaborating with L2 students. She did not believe that her own experiences from studying and growing up in another country influenced the supervision of bachelor’s theses in Norway. By adapting the concept of patient-centered care to student-centered care, the supervisor’s personality and professionalism were considered more significant than cultural background. For supervisors with non-Norwegian backgrounds, it was also pivotal to appear as a role model for L2 students:

“*I think it is so important, in a way, that those of us who come from other countries, who are visible in for example teaching or supervision on bachelor’s thesis, that they see that even if you come from another country, you have managed to earn a doctorate in that country, you know, and worked in a hospital and do exactly the same as any nurse could do, and in addition to that, have built on the academic aspect.*”

(Associate professor 6)

## 4. Discussion

The aim of this study was to explore supervisors’ experiences and perceptions of supervising undergraduate L2 nursing students in their bachelor’s thesis work. The conceptual themes were identified as uncertainty and cultural barriers, cultural considerations, teaching strategies, and supervisor integrity. The themes appeared to align closely with the five identified attributes of cultural humility: openness, self-awareness, egolessness, supportive interaction, self-reflection and critique [23].

Having self-experience from cultural and linguistic adaptation processes seemed to help supervisors to better identify with L2 students and the challenges that they may face in a new context. By considering cultural aspects during supervision sessions, supervisors used this ability as a tool to see beyond surface obstacles such as language difficulties and to discover the individual identities and values in each student. When supervisors were aware of cultural and hierarchical differences in educational systems and addressed the power imbalance between the supervisor and student [15,20], this allowed students to feel more at ease when facing challenges related to “triple socialization”, including language, culture and the academic system [3] (p. 27). Nevertheless, the supervisors did not allude to raising cultural issues as an easy task. Rather, they reported feeling insecure and hesitant, especially when it came to gender disparities. Hence, addressing gender issues may warrant a degree of personal integrity, together with the cultural humility attributes of openness and self-awareness [23].

All supervisors participating in this study stressed the importance of an initial meta-conversation to agree on common goals, ground rules and structure for the supervision period. Variation among supervisors in what they emphasized as important during the supervision period gave insight into the different roles and competencies required of a supervisor. Being a supervisor often implies inherent multiple roles, such as counselors, educators and guides in emotional management [14,17]. Aspiring to make the students feel comfortable and relaxed in supervision settings and allowing for emotional reactions were important for some supervisors. On one hand, this approach aligns with the concept of cultural humility and cultural safety, which does not focus on competence or confidence but rather self-insight about what we do not know about others [18,25]. On the other hand, a certain level of cultural competence may be necessary in a supervisor–student relationship, where the supervisor is expected to demonstrate cultural humility [19]. A majority of the interviewed supervisors were intrigued when being assigned students with Norwegian as their second language, and they confessed to sometimes spending more time on supervision than allocated for each student. Giving L2 students extra time and attention is highlighted as a successful strategy in several studies [7,15] and may be considered an egoless action from the supervisors [23]. Conversely, giving students an extra hour to enhance final results may also be beneficial for supervisors’ reputation and acknowledgment; hence, the motive may potentially be dichotomous.

Some supervisors focused on treating all students equally by complying with pre-set rules and sharing the same information. This may not always be beneficial to L2 students, as it can be an obstacle for recognizing the diversity among students and prohibit success [15]. Rather, supervisors aiming for equity instead of equality would differentiate their teaching methods for better coping and academic success of L2 students [7]. Supervisors with their own experiences from growing up and studying abroad seemed more engaged in explaining the differences in educational practices to the students than supervisors with Norwegian backgrounds. They also tried to encourage students by portraying themselves as good role models. Supervisors being open about their own background may create a feeling of shared understanding in L2 students, although this would depend on supervisor integrity, individual personalities and cultural background [3] (p. 34). Obtaining cultural humility is a lifelong process [20], and individual experience and level of self-reflection among the supervisors will thus vary.

Who we are as human beings and who we are as supervisors is intertwined and reflects our fundamental values, as expressed by one supervisor. This statement may underpin findings of low scores on cultural humility among nursing educators [25]. How can supervisors model the importance of cultural humility to nursing students and inspire them to apply it in their professional practice? Danso’s [19] critique of cultural diversity concepts argues that cultural competence and cultural humility are interchangeable. Both concepts aspire to reach beyond geopolitical borders and languages and include components related to social status, social and ethnic minority groups, interprofessional roles and healthcare provider–patient relationships [19]. This resonates with the position statement of ICN, that nurses are expected to be linguistically and culturally competent to care for all needs of patients and their families [8]. Findings suggest that supervisors within nursing education may benefit from demonstrating cultural humility and cultural competence in a constructive way to empower students and foster such qualities in future nurses. The defined attribute of supportive interaction [23] corresponds to supervisors who described sharing experiences and receiving support from colleagues to improve their guiding practices in support of students. Several supervisors called for a more structured arena where they could raise issues and learn from each other. Facing their own preconceptions and disclosing cultural biases through collegial reflections and constructive critique may strengthen supervisors’ integrity and build upon their personal toolbox of cultural humility and supportive strategies.

### Strengths and Limitations

Although the number of participants in this study could have been higher, the responses from the supervisors provided rich data material. Heterogeneity among the supervisors was considered a strength, as it provided data material from supervisors with various backgrounds, languages and work experiences. Applying different methods for data collection gave the study high validity, as the participants may focus on different aspects of the phenomenon when interviewed individually than in a focus group discussion [33] (p. 71). However, participants may be hesitant to disclose information in a group setting due to group dynamics. There is a possibility that the supervisors who signed up were the most interested in and committed to supervision of L2 students. The supervisors were recruited from one university, although from different campuses. Interviews were conducted by two different researchers, which may have caused a variation in how the interviews were run. Participant verification of transcripts could have strengthened the validation of results. This study is part of a bigger project that also explores students’ perspectives.

## 5. Conclusions

The theoretical framework of cultural humility appears to be a useful guiding tool for supervision of L2 nursing students. The findings are based on a small qualitative sample of supervisors within a Norwegian university context. Nevertheless, the study suggests useful insight and practical guidance to supervisors on what they may consider and reflect upon when guiding L2 students. Common arenas where supervisors can discuss and learn from each other are recommended, in addition to digital resources that can be useful for both students and supervisors. Being culturally humble requires a lifelong learning process of being open to learning, having self-awareness, and showing egolessness and supportive interaction with others, combined with constructive self-reflection and critique. Highlighting and promoting cultural humility in the workplace may enable supervisors to better understand and guide L2 students toward their final goals.

## Figures and Tables

**Table 1:** Example of analysis and interpretation.

Preliminary Themes	Meaningful Units	Coding	Authors’ Reflections	Interpretive Meaning
Supervisors’ knowledge of language and culture	“I have lived many years of my life abroad, so I know something about having to express myself in a language that isn’t my own. So, I think that’s perhaps what I connect with when I meet these students. And sure, it’s about language, but it’s also much about culture, and seeing things in a different way, or having the opportunity to have your own identity […] which you might lose a bit when you have to express yourself in Norwegian.”	Experiences with other cultures Language sensitivity Cultural barriers and gender roles	Are supervisors’ experiences from other cultural settings essential in supervision to L2 students? Equity vs. equality in education: Do L2 students have equal opportunities to succeed as first-language students? Culture sensitivity in communication seems important (language/habits/norms).	Uncertainty and cultural barriers Supervisors are often unsure of what the obstacles are. Self-reflection promotes empathy and sensitivity to cultural barriers and gender roles in the student–supervisor relationship. This may be important in establishing a good supervision environment.

**Table 2:** Participant characteristics.

Number of Participants (7)	Interview/Focus Group Discussion (FGD)	Gender	Age Range	Position	Supervisor Experience	Diverse Cultural Background
3	FGD	Female	50–60 years	Assistant professor	>20 years	No
	FGD	Female	40–50 years	Associate professor	10 years	Yes
	FGD	Male	50–60 years	Assistant professor	>20 years	No
1	Interview	Female	50–60 years	Associate professor	>20 years	Yes
1	Interview	Female	50–60 years	Associate professor	>20 years	No
1	Interview	Female	50–60 years	Associate professor	>15 years	Yes
1	Interview	Female	40–50 years	Assistant professor	<5 years	No

## Data Availability

The data presented in this study are available on request from the corresponding author due to privacy of the participants.

## Public Involvement Statement

No public involvement in any aspect of this research.

## Guidelines and Standards Statement

This manuscript was drafted against the COnsolidated criteria for REporting Qualitative research (COREQ).

## Abbreviations

The following abbreviations are used in this manuscript:

L2	Second Language
SIKT	Norwegian Agency for Shared Services in Education and Research
FGD	Focus Group Discussion
STC	Systematic Text Condensation
ID	Interpretive Description

## Appendix A. Interview Guide for Supervisors

Project: Multilingualism as a Resource in Thesis Writing for Students with Norwegian as a Second Language

### Introduction to the Topic and Project

Problem statement: How can supervisors utilize knowledge of language and culture as tools in supervision of bachelor’s thesis for second-language nursing students?

Questions

1. What prior knowledge do you have about culture and language that can help you in supervision situations when students do not have Norwegian as their first language?
  - Is there anything that you feel you lack knowledge about?
2. What is your perspective on the importance of supervisors having knowledge and experience in cultural understanding and language when guiding multilingual students?
  - Do you have examples of recommendations or strategies that could be useful for other supervisors?
  - Do you have examples of recommendations or strategies that could be useful for the students?
3. Can you tell us about a bachelor’s thesis that you have supervised in the nursing program, where the student(s) had Norwegian as a second language?
  - How do you experience supervising students who have Norwegian as a second language?
  - How would you describe your supervision style?
  - How do you supervise regarding language-related challenges?
  - How do you supervise concerning the academic content of the thesis?
  - What is important to you when you supervise students?
  - What do you find is important for the students?
  - What is it like to supervise students within the framework of this university?

Possible other follow-up questions according to the development of the interview or the discussion in the group.
